# Operative Use of Thoracic Ultrasound in Respiratory Medicine: A Clinical Study

**DOI:** 10.3390/diagnostics12040952

**Published:** 2022-04-11

**Authors:** Gino Soldati, Renato Prediletto, Marcello Demi, Stefano Salvadori, Massimo Pistolesi

**Affiliations:** 1 Ippocrate Medical Center, 55032 Lucca, Italy; soldatigino@yahoo.it; 2 Pulmonology Unit, Fondazione Toscana Gabriele Monasterio and National Research Council, 56124 Pisa, Italy; predile@ftgm.it; 3 Department of Bioengineering, Fondazione Toscana Gabriele Monasterio, 56126 Pisa, Italy; 4 Clinical Physiology Institute, National Research Council, 56124 Pisa, Italy; stefsa@ifc.cnr.it; 5 Department of Respiratory Disease, University of Firenze, 50121 Firenze, Italy; massimo.pistolesi@unifi.it

**Keywords:** diagnosis, lung, respiratory medicine, sonography, ultrasound

## Abstract

For over 15 years, thoracic ultrasound has been applied in the evaluation of numerous lung diseases, demonstrating a variable diagnostic predictive power compared to traditional imaging techniques such as chest radiography and CT. However, in unselected pulmonary patients, there are no rigorous scientific demonstrations of the complementarity of thoracic ultrasound with traditional and standardized imaging techniques that use radiation. In this study 101 unselected pulmonary patients were evaluated blindly with ultrasound chest examinations during their hospital stay. Other instrumental examinations, carried out during hospitalization, were standard chest radiography, computed tomography (CT), and, when needed, radioisotopic investigation and cardiac catheterization. The operator who performed the ultrasound examinations was unaware of the anamnestic and clinical data of the patients. Diffuse fibrosing disease was detected with a sensitivity, specificity and diagnostic accuracy of 100%, 95% and 97%, respectively. In pleural effusions, ultrasound showed a sensitivity, specificity and diagnostic accuracy of 100%. In consolidations, the sensitivity, specificity and diagnostic accuracy were 83%, 98% and 93%, respectively. Low values of sensitivity were recorded for surface nodulations of less than one centimeter. Isolated subpleural ground glass densities were identified as White Lung with a sensitivity of 72% and a specificity of 86%. Only the associations Diffuse ultrasound findings/Definitive fibrosing disease, Ultrasound Consolidation/Definitive consolidation and non-diffuse ultrasound artefactual features/Definitive vascular pathology (pulmonary hypertension, embolism) were statistically significant with adjusted residuals of 7.9, 7 and 4.1, respectively. The obtained results show how chest ultrasound is an effective complementary diagnostic tool for the pulmonologist. When performed, as a complement to the patient’s physical examination, it can restrict the diagnostic hypothesis in the case of pleural effusion, consolidation and diffuse fibrosing disease of the lung.

## 1. Introduction

Thoracic ultrasound (US) is a recently developed diagnostic tool [1,2]. Most literature deals with cardiogenic pulmonary edema, ARDS, bronchiolitis in children and pneumonia in adults [3,4,5]. Few studies, however, are aimed at studying focal, multifocal and diffuse lung diseases providing information on pleural signs, pulmonary artifacts morphology and pathological changes such as nodules and consolidations along with their surface distribution. Moreover, many works in the past based their attention on the artifacts numerical estimate for scoring the severity of a pathological state, but, to our knowledge, a rather limited number of clinical and technical papers investigated the meaning of their morphology [6,7,8,9,10,11,12,13].

This limits the ability to characterize and differentiate cardiac from pleuro-pulmonary pathological conditions, especially when these disorders overlap. It is worth noting that both pulmonologists and cardiologists hold the same view as regards thoracic ultrasound semeiotics in that it is mostly based on the classical “wet and dry” lung dichotomy. This work aims to clarify the diagnostic role of thoracic ultrasound on an unselected population of patients with pulmonary diseases and to provide clinical support to the hypotheses on the genesis of pulmonary ultrasound artifacts recently suggested [9,14,15]. The following points will be discussed: (1) the practical role of thoracic ultrasound, seen as a specific tool of the pulmonologist for “visiting” the patient and to restrict the field of the diagnostic options; (2) the associations between artefactual and anatomic ultrasound signs and CT findings; (3) the semeiotics and meaning of pulmonary artifacts based on the potential genesis of pleuro-pulmonary signs.

## 2. Materials and Methods

### 2.1. Clinical and Instrumentale Evaluation

We evaluated 101 subjects who had been admitted from November 2017 to December 2018 to the Pulmonary Medicine Unit of the Gabriele Monasterio Tuscany Foundation of Pisa, with 103 ultrasound chest examinations. Patients were studied in the Pulmonology Unit, either as an emergency or as a programmed hospitalization, on the basis of their pathological conditions and/or the onset of respiratory symptoms, such as recent dyspnea and chest pain in order to complete a diagnostic and therapeutic work-up. The exclusion criteria were pulmonary or cardiac acute decompensation during the evaluation, acute respiratory distress syndrome, heart and valvular diseases, fever, myocardial infarction, pneumothorax and thoracic trauma. All other pulmonary pathological conditions in patients older than 18 years were included. During the ultrasound examination all patients were in stable clinical conditions.

Every patient was informed in advance about the research content, and they provided signed informed consent. The investigation was authorized by the Institute’s Ethics Committee CEAVNO (study number 1089, approved on 30 January 2017). All patients completed a thorough respiratory and cardiac evaluation by spirometry, gas exchange, single breath diffusion capacity, chest X-ray, CT scan, and perfusion lung scan evaluation. Chest CT findings were considered the gold standard for the anatomic evaluation of pleura and lung parenchyma. All chest high-resolution CT examinations were performed at our hospital’s Department of Radiology using standard protocol. No intravenous contrast material was administered. CT images were evaluated by experienced radiologists and by a chest physician (RP) who is board certified in Radiology, using the traditional radiological lexicon. Discordance between radiologists and the chest physician were resolved by a consensus CT reading. The final diagnosis was drawn up by the chest physician following a thorough examination of the clinical and instrumental data.

**Figure 1:**
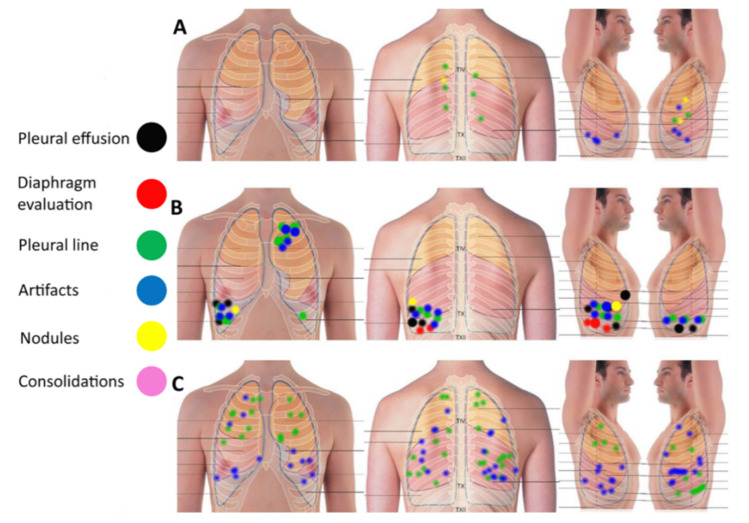
Example of the location of ultrasound (US) findings on the thoracic surface through a graphic scheme with differently colored landmarks, to obtain a superficial topography comparable to CT images. Three schemes of three different patients are shown in A, B and C.

The ultrasound examination was carried out by a single operator (GS) with more than twenty years’ experience in chest ultrasound. The operator who performed the ultrasound examinations was unaware of the anamnestic and clinical data of the patients. We used a Toshiba Aplio V machine, equipped with convex (3.5–5 MHz) and linear (5–9 MHz) probes. Harmonic imaging was not used. With patients in a sitting position, posterior, basal, paravertebral and apical scans were obtained. Frontal and lateral scans, including supraclavicular regions were obtained in a supine position. Findings such as artifacts, consolidations, nodules, effusions, diaphragmatic position and kinetics were defined according to semeiotics described in the literature [1,2,3,5,8]. Table 1 lists the findings and the possible generic diagnoses which can be expected with the ultrasound and CT examination, respectively. These characteristics were recorded and stored in a database. The location of ultrasonographic findings on the thoracic surface was described for each patient through a graphic scheme with differently colored landmarks (Figure 1), to obtain a topography comparable to CT images.

Pleural effusions were identified on the basis of their classical ultrasound signs. Patients with pleural effusion were not considered as belonging to a specific subgroup since the effusion may be associated with different pathological conditions.

The distinction between pneumogenic and cardiogenic artifacts was made according to their structure and relative distribution and homogeneity along the pleural line [16]. Only the brightest artifacts with characteristics of full screen extension, pleural-point origin with or without internal modulation were considered “B-lines” [16,17]. All other artifacts were generically referred to as “vertical artifacts”. White Lung [9,16] was identified as a focal or multifocal artifact characterized by an undifferentiated echogenic background, with the absence of A-lines and without evidence of vertical artifacts.

The presence of subpleural nodularities (consolidations with relatively defined contours with maximum size less than or equal to 1 cm) was simply reported. Subjects with dominant consolidations, single or multiple, mono or bilateral consolidations with a diameter greater than 1 cm, were assigned to a specific subgroup.

The term hypermirror, not mentioned in the literature, was arbitrarily introduced. It indicates a finding that covers at least a third of a lung field characterized by a regular pleural line and by the absence of vertical artifacts and B-lines. Its typical appearance is that of a pronounced mirror effect with an artefactual replica of the pleural line (two replicas at least). In presence of emphysema, the air content of the subpleural lung layers increases and, as a consequence, the reflection coefficient at the pleural plane also increases giving rise to more pronounced replica and mirror effects. Moreover, a suboptimal configuration of the diaphragm occurs in emphysema and the demand imposed by the pathology on this muscle increases due to both hyperinflation and air-flow obstruction. In certain types of emphysema (e.g., panlobular emphysema), a lower position and reduced dynamics of the hemidiaphragms is also expected. The hypermirror finding, along with both an objective reduction of the maximum diaphragmatic excursion (less than 3.5 cm in the middle axillary to the maximal inspiration in sitting position) and lowered hemidiaphragms, was considered as a sign of emphysema.

Based on the results of the ultrasound examination, the sonographer blindly assigned each subject to one of five groups (Table 2). This classification is based on the recognized non-specificity of many ultrasound signs. Therefore, it is more useful to refer to ultrasound patterns (typology and association of signs, topography, extension) rather than to single uncoordinated signs. While the terms “consolidation” and “nodule” have a real meaning (tissue without air), variable configurations of the artifacts assume a diagnostic power only through their distribution (focal, multifocal and diffuse). The groups were as follows:

Group 1: No pathological findings.

Group 2: Limited findings. Monofocal or multifocal artifacts, with or without nodularity (small consolidations abutting the pleura with a diameter ≤ 1 cm). This group was characterized by focal or multifocal findings whose characteristics did not allow us to hypothesize a specific pathological condition.

Group 3: Diffuse findings. Pathological artefactual signs (B-lines and/or vertical artifacts) spread bilaterally over two thirds of the lung fields.

Group 4: Consolidations. Single or multiple, mono or bilateral consolidations with a diameter > 1 cm, showing variable conditions of internal ventilation. The presence of vertical artifacts or perilesional White Lung was considered a related finding. Patients from other groups may present consolidations or nodules, but non-dominant with respect to other findings.

Group 5: Non-diffuse features. Presence of pathological artifacts, with or without nodularity (consolidations < 1 cm), configuring a pathological picture which extended up to two thirds of a pulmonary field.

### 2.2. Data Analysis

The anthropometric and clinical data of the patients including history, evolution and outcome US, CT findings, and other instrumental findings were coded and saved in a database. Descriptive statistics were expressed as mean +/− SD or as numbers with percentages. CT images with radiological descriptions were stored together with video clips from the ultrasound examinations and with the surface maps (Figure 1). The sonographer (GS) was unaware of any clinical and instrumental data and had access only to the ultrasound database. Both the clinical management and the organization and visualization of the clinical and CT database was assigned to and carried out by a single operator (RP). The attribution of patients to the US groups, described in Section 2.1, was carried out independently and blindly by the sonographer. Sonographic signs were interpreted and classified by the sonographer while the clinician was precluded from accessing the ultrasound database.

The primary endpoint of this study was to investigate the role of pattern-based chest ultrasound on an unselected population of patients admitted to a pulmonology department and undergoing a blinded ultrasound examination.

The secondary endpoint was to evaluate potential associations between established ultrasound findings and CT, and to discuss the role of selected findings as acoustic information related to the subpleural structure of the lung as seen in CT [9].

In order to evaluate the endpoints in the simplest way, two different evaluations were made: 

(a) After a complete diagnostic work up, the subjects were divided into four groups of definitive pathologies: consolidative pathology, diffuse fibrosing disease, COPD, and vascular pathology (pulmonary hypertension, embolism). Descriptive analysis was performed and expected frequencies were reported within the contingency table of the distribution of “Group” by “Definitive pathology”. To evaluate the structure of the association of the two variables, adjusted residuals were calculated for each cell, and *p*-values were adjusted as described by Beasley and Schumacker [18] in order to take multiple comparisons into account. A *p*-value < 0.00256 was considered statistically significant.

(b) Sensitivity and specificity of the ultrasound examination findings (vertical artifacts and B-lines, White Lung areas, consolidations, nodules) were calculated versus interstitial changes (septal and or reticular), ground glass density, consolidations and nodularities, respectively, using CT as the reference test. The specificity of the tests was defined as TN/(TN + FP), the sensitivity as TP/(TP + FN), the positive predictive value (PPV) as TP/(TP + FP), and the negative predictive value (NPV) as TN/(TN + FN).

## 3. Results

A total of 101 subjects, 55 males and 46 females ranging in age from 33–90 years, with an average age of 71, were evaluated with 103 ultrasound acquisitions. Two subjects had two acquisitions each. The chest ultrasound was carried out within 4 days of admission. Each ultrasound examination lasted no more than 15 min (range 8–15 min). A chest CT was carried out within 2 days of the ultrasound examination. The patients’ subgroups are illustrated in Table 2. Seven subjects were free from pathological ultrasound findings and their final diagnoses are listed in Table 3. Table 4 illustrates the associations between the groups of the enrolled patients and the four groups of final pathologies described in Section 2.1. Only the association Diffuse findings/Fibrosing disease, Consolidation/Consolidation and Non-diffuse features/Vascular pathology (pulmonary hypertension, embolism) were statistically significant with adjusted residual of 7.9, 7 and 4.1, respectively.

Thirty-nine pleural effusions were identified in 28 subjects (27.1%), 19 of which were minimal effusions (48.7%), i.e., only visible in the lateral costophrenic sinus. The effusions were bilateral in 11 patients (39.2% of the subjects with pleural effusion). All subjects without significant lung findings (Group 1) did not show any effusion. Among those with limited or monofocal findings (Group 2), diffuse findings (Group 3), consolidations (Group 4) and non-diffuse artefactual syndrome (Group 5), effusions were detected in 6, 2, 14 and 6 patients (17%, 12%, 64% and 17% of the subjects of each group), respectively. In pleural effusions, ultrasound showed a sensitivity, specificity and diagnostic accuracy of 100%.

Vertical artifacts and B-lines were detected in patients of all groups except in patients in Group 1. Furthermore, when considering the entire population studied, while the artifacts (B-lines and vertical artifacts) were present in 78 patients (75.7%), B-lines were present in 38 subjects only (36.8%). Only one patient showed B-Lines exclusively without vertical artifacts. All the other subjects with B-lines also showed vertical artifacts. Vertical artifacts in subjects without B-lines were present in 40 patients (52.2% of the entire population with artifacts).

B-lines were detected in all six patients whose CT findings were compatible with subpleural hydrostatic septal thickening. The sensitivity and specificity of B-lines in subjects with pulmonary edema was 100% and 68% respectively, as 31 subjects showed B-lines in the absence of CT findings of subpleural septal thickening.

Consolidations were detected in CT in 34 subjects and all subjects in Group 4 showed confirmation of the findings (Table 3). Six patients in Group 2 with CT consolidations turned out to be ultrasound false negative: three patients showed lesions which did not reach the pleura (two neoplasms and one inflammatory lesion) and three subjects showed findings which were compatible with atelectasis. Among the detected consolidations, a total of 11 neoplasms were diagnosed (10 lung tumors and one mesothelioma), 9 of which were visible on the ultrasound images. Mesothelioma was correctly detected.

In consolidations, the sensitivity, specificity and diagnostic accuracy were 83%, 98% and 93% respectively. Four of the six false negatives occurred in patients with central and non-subpleural lesions which were not visible on the ultrasound images. Sensitivity increases to 88% by excluding these patients.

Nodules were detected in 23 patients. CT showed superficial nodules in 55 patients. Sensitivity and specificity of ultrasound was 45% and 78%, respectively.

White Lung was detected in all groups except in Group 1. In order to compare isolated White Lung with CT ground glass findings, we selected those subjects who showed the White Lung artifact only (subjects without pleural effusion, consolidations or nodulations). Subjects with CT findings of ground glass not surfacing the pleura were not considered due to the limitation of ultrasounds to detect them. Among the 23 subjects whose CT showed subpleural isolated ground glass, 18 of them had corresponding areas of ultrasound White Lung. Sensitivity and specificity of ultrasound in detecting superficial ground glass were 72% and 86%, respectively. Positive and negative predictive values were 76% and 94%.

Of the forty-four subjects who were diagnosed with COPD or asthma, only 2 subjects did not show ultrasound signs. Forty-two subjects showed a variety of signs and were therefore included in the other groups (58.3% in Group 2, 29.4% in Group 3, 39.1% in Group 4 and 26.3% in Group 5).

The tree-in-bud CT pattern was detected in 13 subjects belonging to Groups 2 (7 subjects), 3 (2 subjects), 4 (2 subjects) and 5 (2 subjects), respectively. Among them, 4 subjects showed focal B-lines and vertical artifacts, 8 patients showed nodulations or microconsolidations.

The association of a hypermirror pattern with lowered hemidiaphragms and a reduction of the maximal diaphragmatic inspiratory excursion, showed a sensitivity, specificity and diagnostic accuracy of 45%, 96% and 86%, respectively, for the identification of subjects with pulmonary emphysema detected with CT.

**Figure 2:**
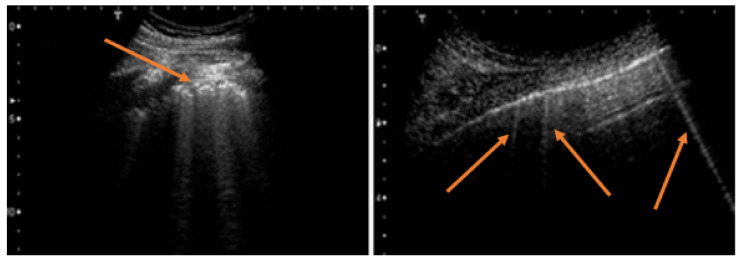
The image on the left shows vertical artifacts in a subject with idiopathic pulmonary fibrosis with a strongly irregular pleural line (red arrow). The image on the right shows B-lines (red arrows) with a regular pleural line in a subject with heart failure.

**Figure 3:**
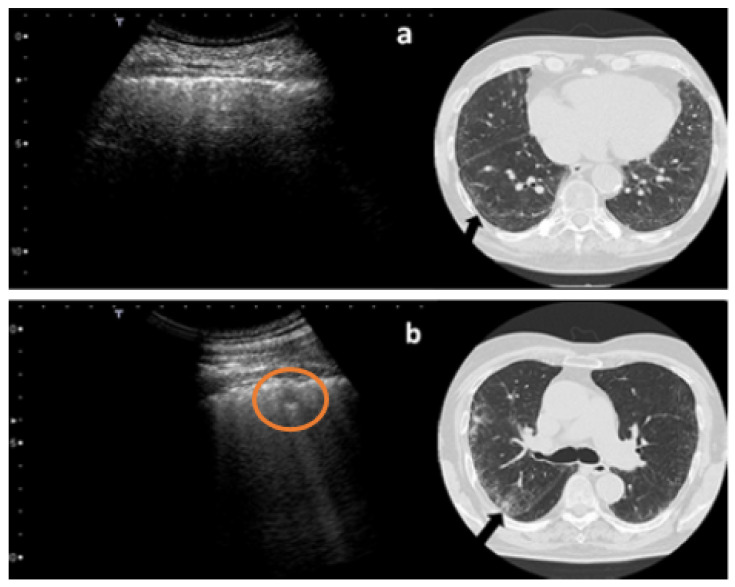
Ultrasound and CT scans of two patients with fibrosing lung disease. The black arrows indicate the position of the probe on the chest. The two CT and ultrasound scans are coplanar. In (**a**) a minor fibrotic involvement is present while in (**b**) a small nodulation (red circle) is evident.

**Figure 4:**
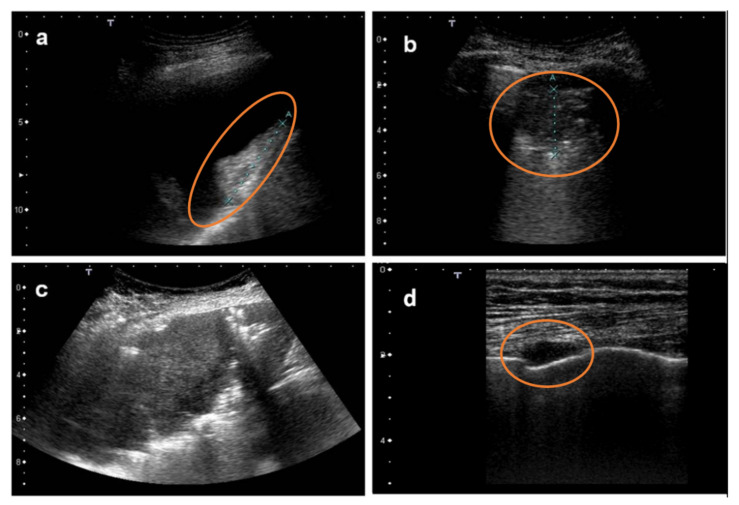
Image (**a**) shows an ultrasound scan of a patient with large pleural effusion and a coarse vegetation on the diaphragmatic surface (metastatic carcinoma in red circle). Image (**b**) shows lung consolidation with poor ventilation (pneumonia in red circle). Image (**c**) shows a large lung consolidation (lung cancer). Image (**d**) shows pleural neoplasia (mesothelioma in red circle).

**Figure 5:**
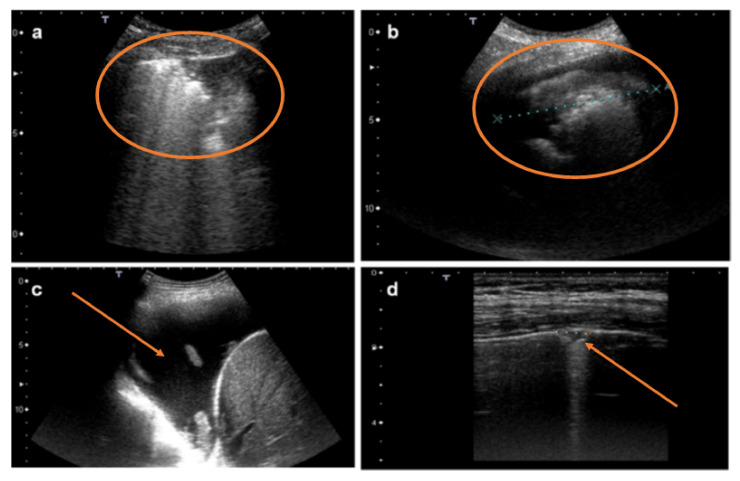
Image (**a**) shows lung consolidation with associated artifacts in a red circle (pneumonia). Image (**b**) shows lung consolidation with associated pleural effusion (lung cancer in red circle). The red arrow in image (**c**) shows a large finely corpuscular pleural effusion (mesothelioma). The red arrow in image (**d**) shows a single nodule emerging on the pleural surface with a regular pleural line.

**Figure 6:**
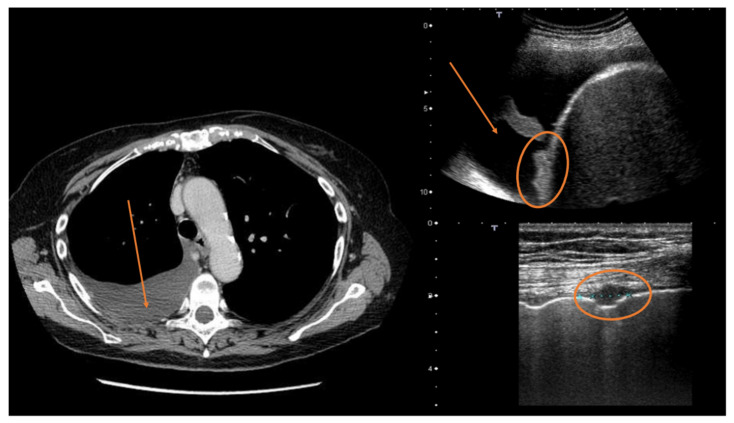
Ultrasound scans of a patient with mesothelioma. On the left, the CT scan shows only pleural effusion (red arrow). The upper right ultrasound scan clearly shows the pleural effusion (red arrow) and diaphragmatic nodulations (red circle). The lower right ultrasound scan shows a parietal pleural nodule (red circle).

Figure 2, Figure 3, Figure 4, Figure 5 and Figure 6 show some examples of the main echographic signs which have been evaluated in this study.

## 4. Discussion

Pleuro-pulmonary ultrasound is widely reported in scientific literature. Although ultrasound semeiotics of pleural effusion and pulmonary surface consolidations are widely described and accepted [2], the sonographic patterns in non-consolidated pathological conditions are still under investigation and, in general, limited to the description of A-lines and (in quantitative terms) B-lines [19,20].

Most of the B-line literature regards cardiology patients with heart failure. In this context, the results are generally unique, and the B-lines were found to correlate with the increase of the extravascular pulmonary water and the severity of the pathology.

Recently, the increased interest in the ultrasound signs in ARDS, interstitial pulmonary diseases and COVID-19 pulmonary involvement has raised the need for a better classification of the artifacts to increase their specificity [15,16,21,22]. Some observations point out the great variability of the artifacts and suggest the use of their morphology and distribution pattern for a preliminary tissue characterization of the lung surface [10,11,23].

However, most of the published studies regard the settings of Emergency Medicine, Intensive Care, Cardiology, Pediatrics and Rheumatology. Only a few studies are available on Pulmonary Medicine patients [6,22,24]. Many patients have been selected according to specific pathological conditions (pulmonary edema, ARDS, pneumonia, bronchiolitis, collagen diseases) and to single sonographic findings. A few publications address the application of ultrasounds as an initial blind test in patients with the several pleuro-pulmonary pathological conditions encountered in a Respiratory Medicine Unit.

This paper evaluates an unselected series of non-critical patients admitted to a Pneumology Unit, and endeavors to represent the clinical workload of a pulmonologist’s daily diagnostic activity. The great majority of our patients had more than one pathological condition, even though their prevailing clinical condition was of pulmonary origin.

By fixing an initial pattern evaluation of ultrasound findings (patient groups 1 to 5), it is possible to narrow down the diagnostic possibilities. This is particularly true for diagnosing consolidations and fibrosing lung diseases, appearing as deaerated lesions and diffuse pneumogenic artifacts, respectively [6,22].

Conversely, the absence of ultrasound signs, or the presence of artifacts with limited monofocal or multifocal distribution, with or without nodularity or non-diffuse artefactual features, remains non-specific outside a clinical context [25].

The association between non-diffuse features and vascular pathology (pulmonary hypertension, embolism) achieves statistical significance, but does not find a clear pathogenetic explanation. This probably merely reflects the non-specificity of non-diffuse pulmonary artifacts or the coexistence of multiple pathologies in the same patient.

When chest CT is used as a reference standard, pleuro-pulmonary sonography shows high accuracy for the detection of pleural effusion and pneumonia (sensitivity and specificity of 93–94% and 96–98% respectively) [5,26,27,28,29].

Our experience agrees with these data. The best diagnostic accuracy of ultrasound, compared with a set of diagnostic procedures including CT, was obtained for the detection of pleural effusions. Moreover, ultrasound is a useful tool for the evaluation of lung consolidations with a sensitivity of 83% and a high specificity (98%).

Its role is confirmed in pneumonia, atelectasis, inflammatory consolidations and neoplasms when they surface the pleura. 11 cases of pneumonia in 12 patients (91.6%) were correctly identified. Overall, 9 neoplastic lesions out of a total of 11 neoplasms (10 pulmonary and one pleural neoplasia) were detected by ultrasound since two of them (two pulmonary neoplasms) did not surface the pleura. Five neoplastic subjects presented pleural effusion that was fibrinous in two cases and corpuscular in one. Atelectasis was diagnosed by ultrasound in 9 cases, and all were confirmed by CT. Atelectasis was associated with neoplasia in two patients and to effusion or hypoventilation in the remaining 7 patients.

The performance of the US for cancer detection is limited only because US can show only the neoplasms which surface the pleura. The same problem exists for the definition of many pulmonary nodularities.

Lung artifacts (vertical artifacts and B-lines) were the most frequent pathological findings observed [15] (Figure 1). This study confirms the distinction described in literature between pneumogenic and cardiogenic artifacts based on their distribution and on the characteristics of the pleural line [16]. Artifacts in focal position and/or limited in their representation (Groups 5 and 2 respectively) appear non-specific outside a clinical context and generically indicate a pneumogenic origin, often related to COPD/asthma.

Many studies in literature have addressed the diagnostic role of artifacts. The Blue Protocol [30] applied to patients with acute dyspnea in the Emergency Room showed a sensitivity of 88% and a specificity of 96% of B-lines for the diagnosis of acute cardiogenic pulmonary edema. Moreover, if compared to chest radiography, ultrasound shows greater sensitivity in differentiating the dyspnea due to cardiogenic pulmonary edema from the dyspnea due to exacerbation of chronic obstructive pulmonary disease [31,32,33]. In a recently published review of patients with acute heart failure [34], ultrasound showed better diagnostic accuracy for pulmonary edema than chest radiography with a sensitivity of 88% and a specificity of 90%, respectively.

Vertical artifacts and B-lines report a non-consolidating increase in subpleural density which, through an interstitial expansion, opens acoustic channels which allow the transmission of acoustic energy to acoustic traps. Pairs of channels and traps with different shapes and sizes highlight specific harmonics of the pulse power spectrum which are subsequently represented visually by the scanner as artifacts with variable morphologies [14,15,20].

The evidence that three quarters of our patients showed pneumogenic artifacts associated with increased subpleural CT density (as in 17 cases with diffuse interstitial pathology) increases the plausibility of the hypotheses set out above. On the contrary, 23 subjects without vertical artifacts and B-lines had final diagnoses which excluded subpleural hyperdensity (12 patients with COPD/emphysema with pulmonary hypertension, 2 with heart disease without pulmonary congestion, 2 with pulmonary embolism, 3 with neoplasia and 1 with collagen disease without pulmonary involvement).

Vertical artifacts and B-lines have been described as being associated with consolidations [35]. Our findings indicate that neoplastic growth, contrary to pneumonia, can produce consolidations which are not surrounded by vertical artifacts or B-Lines and, on the other hand, that B-lines are more frequent in inflammatory consolidations where inflammatory edema is present. An inflammatory consolidation is usually surrounded by edematous tissue and, in this case, artifacts spreading from the borders of the consolidation are visible. This characteristic is usually absent or less evident at the borders of the neoplastic processes.

Six patients had ultrasound signs compatible with cardiogenic lung interstitial edema despite the absence of echocardiographic signs of heart decompensation. These subjects had B-lines associated with other vertical artifacts, which were probably justified by the concomitance of primary pulmonary pathology (COPD, interstitial lung disease and pneumonia). The wide distribution of the B-lines in the other groups and in subjects without heart failure testifies the low specificity of this finding for pulmonary edema (68%) which is lower than that mentioned in the literature in selected cases with low or no prevalence of primary lung disease [36]. However, the sensitivity of B-lines for pulmonary edema is 100%.

The presence of B-lines is not strictly related to cardiogenic components, and in our opinion, the presence of diffuse B-lines should be considered as a simple indication for the evaluation of the systolic and diastolic function of the left ventricle.

Different groups of patients give rise to different patterns and may also require different study protocols [37]. Different approaches to the patient may be required by critical subjects in intensive care or in the emergency room or by subjects with chronic pleuro-pulmonary pathologies. The pediatric and neonatal setting, although exploiting investigation methodologies and semiotics which are the same as those of the adult, may require specific methodologies. Finally, the recent COVID-19 pandemic, while validating some hypotheses on the origin of the artifacts, has also required special methods of investigation [38,39].

Vertical artifacts are observed in lung interstitial diseases. In these pathologies, the interstitium undergoes an irregular and dense expansion. Therefore, variable, irregular vertical artifacts arising from a blurred pleura are expected because of the presence of many different and irregular acoustic channels [11,14,15]. Diffuse fibrosing disease was detected with a high sensitivity and specificity. According to the final outcome, 15 subjects in group 3 (88.2%), and one subject in Group 5 were affected by interstitial fibrosis.

It has been speculated that the sonographic artifact called “White Lung” may represent a subpleural condition of homogeneous increased density that may strictly anticipate and progress to consolidation [15]. The most probable hypothesis of its genesis, echoes radiated by a subpleural distribution of small scatterers [9], suggests a correlation between the sonographic “White Lung” and the ground glass densities on CT. In our experience, the positive predictive value of US to detect White Lung was low (76%) compared with the detection of ground glass by CT, due to the limitation of ultrasound detection of artifacts (including White Lung) beyond the surface of the lung.

While classical teaching states that subjects with COPD and asthma do not show ultrasound changes [30,33], we rarely observed subjects with COPD or asthma without aspecific mono or oligofocal sonographic findings. Similar conclusions can be drawn for subjects with the tree-in-bud CT pattern. Patients with COPD, asthma and the tree-in-bud CT pattern can show mono or oligofocal findings with non-specific characteristics, probably related to hypoventilation, fibrosis, micro- consolidation or aspecific nodularities.

Finally, simple observations related to the hemidiaphragms with the evidence of hypernormal lung regions (hypermirror effect) can characterize subjects with emphysema. Preliminary results support this hypothesis. However, the role of ultrasound in emphysema has not been previously described and further studies are necessary to confirm this suggestion.

Our study has some limitations. The heterogeneity of the diseases is a limit to the definition of the ultrasound diagnostic accuracy for the single pathologies. Furthermore, the diagnostic framework with ultrasound was carried out by a single expert operator, and our results cannot be extended to all pneumology centers in the absence of skilled physicians.

## 5. Conclusions

Chest ultrasound is a safe and effective complementary diagnostic tool for the pulmonologist, and its role in an initial evaluation in unselected respiratory patients admitted to a Hospital Pulmonary Medicine Unit is important. The fact of not using ionizing energies and the simplicity of the US examinations makes this diagnostic tool directly accessible to all physicians in every situation. The high sensitivity of US to density increases of the lung surface is surely its most important strength. Another strength of US is its diagnostic accuracy in pleural pathology and lung consolidations. In these cases, a greater sensitivity than the chest X-ray is demonstrated. However, we believe that other applications may benefit from thoracic ultrasound. These include the screening and monitoring of diffuse interstitial pathologies (which are often imperceptible in chest X-ray images) and the diagnosis and monitoring of viral pneumonia. Finally, the interpretation of pulmonary hyperinflation and specifically pulmonary emphysema is a completely unexplored field. Conversely, the weaknesses of pulmonary ultrasound regard the limited field of view of the individual scans, the existence of areas that cannot be explored, and the impossibility of detecting lesions that do not affect the pleural surface. Moreover, the specificity of B-lines is still under study but is believed to be low if they are not included in a clinical evaluation of the patient. In patients with pleural effusion, consolidations, and diffuse interstitial diseases ultrasound can sensitively identify the generic pathology, its location and characteristics, thus achieving a higher diagnostic yield when compared with CT. In diffuse interstitial diseases the main factor favorably affecting the diagnostic yield is the subpleural location of the tissue hyperdensities, appearing in ultrasound as pneumogenic vertical artifacts. Our observations confirm the hypothesis that vertical artifacts (to which the known B-lines belong) are an extremely varied category of artifacts and we believe that an analysis of the latter, based on their visual characteristics and distribution, may be more useful than a mere quantitative count. In particular, recent evidence of the relationships between the internal structure of the vertical artifacts and the geometry of the acoustic trap/channel systems which have generated the artifacts opens new perspectives for increasing their specificity [9,10,11,15]. However, independently of its specificity, the artefactual ultrasound information of the lung must always be rationally integrated in a multiple district and multi-instrumental clinical context.

## Figures and Tables

**Table 1:** Observed/registered findings and possible generic diagnoses expected with the ultrasound and CT examination.

Ecographic Findings and Assumed Diagnosis	CT Findings and Assumed Diagnosis
Vertical artifacts	Septal thickening
B-lines	Reticular thickening
Hypermirror	Consolidation
Consolidation	Tree-in-bud
White Lung	Ground glass
Pleural effusion	Pleural thickening
Micronodules	Pleural effusion
Pleural thickening	Surface micronodules
Diaphragm kinetics	
	
Emphysema	Lung cancer
Focal interstitial syndrome	Pleural cancer
Diffuse interstitial syndrome	Diffuse interstitial syndrome
Cardiogenic contribution	Cardiogenic pulmonary edema
	Emphysema

**Table 2:** Characteristics of patient’s subgroups.

Group	Male	Mean Age (y+/−sd)	Female	Mean Age (y+/−sd)	Total
No pathological findings	3	66+/−10	4	53.5+/−8.3	7
Limited findings	18	70.8+/−7.4	18	71.7+/−12.8	36
Diffuse findings	11	75.9+/−8.9	6	72.8+/−9.6	17
Consolidations	16	73.6+/−11.4	8	76.5+/−4	24
Non-diffuse features	9	73.2+/−8.4	10	63.6+/−16.3	19

**Table 3:** Final diagnosis in seven patients free from pathological ultrasound findings.

Patient	Sex	Final Diagnosis
1	F	Pulmonary Hypertension
2	F	Dyspnea in bronchial hyperreactivity
3	F	Syncope in a patient with susceptibility to the development of vaso-depressant syncopal episodes in response to prolonged orthostasis.
4	M	Type 1 respiratory failure secondary to right-left shunt from patent foramen ovale type ostium secundum
5	M	Type 1 respiratory failure of a nature to be determined with right saphenous small thrombophlebitis
6	F	Bronchitic exacerbation in patient with bronchiectasis
7	M	Respiratory insufficiency in pulmonary consolidations under diagnostic definition, resolved at the time of examination

**Table 4:** Associations between the groups of the patients according to the initial sonographic selection and the four groups of final pathologies described in methods.

Group		Definitive Pathology				Total
		Consolidation	COPD	Chronic Fibrosing Disease	Vascular Pathology	
Consolidations	Count	22	1	0	1	24
	Expected Count	7.9	6.3	3.5	6.3	
	% Within Group	91.7%	4.2%	0.0%	4.2%	
	Adjusted residual	7.0	−2.8	−2.3	−2.8	
Diffuse Findings	Count	0	3	13	1	17
	Expected Count	5.6	4.5	2.5	4.5	
	% Within Group	0.0%	17.6%	76.5%	5.9%	
	Adjusted residual	−3.2	−0.9	7.9	−2.1	
Limited Findings	Count	8	15	2	11	36
	Expected Count	11.9	9.4	5.2	9.4	
	% Within Group	22.2%	41.7%	5.6%	30.6%	
	Adjusted residual	−1.7	2.6	−1.9	0.7	
No pathological ultrasound findings	Count	1	4	0	2	7
	Expected Count	2.3	1.8	1.0	1.8	
	% Within Group	14.3%	57.1%	0.0%	28.6%	
	Adjusted residual	−1.1	1.9	−1.1	0.1	
Non-diffuse features	Count	3	4	0	12	19
	Expected Count	6.3	5.0	2.8	5.0	
	% Within Group	15.8%	21.1%	0.0%	63.2%	
	Adjusted residual	−1.8	−0.6	−2.0	4.1	
Total	Count	34	27	15	27	103
	% Within Group	33.0%	26.2%	14.6%	26.2%	
